# *Kamzori*: Aging, Care, and Alienation in the Post‐pastoral Himalaya

**DOI:** 10.1111/maq.12707

**Published:** 2022-04-29

**Authors:** Nikita Simpson

**Affiliations:** ^1^ Department of Anthropology London School of Economics and Political Science

## Abstract

As the Gaddi community of Himalayan India transition from agro–pastoralism to waged labor, configurations of kinship and care have shifted. Such shifts have introduced relational tensions, especially between elderly women, who have labored in the house and fields, expecting care in old age, and younger generations, who experience their own pressures of class aspiration. This article examines how the myriad tensions of the post‐pastoral economy are experienced in the bodies of elderly women. It presents insights on *kamzori*, bodily weakness that is experienced by women who feel that their contribution of labor and care is unreciprocated by their kin or wider milieu. It recuperates alienation as a concept that captures distressed social relations. Alienation might be used by anthropologists studying aging, care, and debility to envisage the body in scalar relation to people, things and places, and illness or distress as disruption of such relations. *[weakness, aging, care, gender, alienation]*

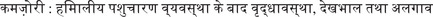

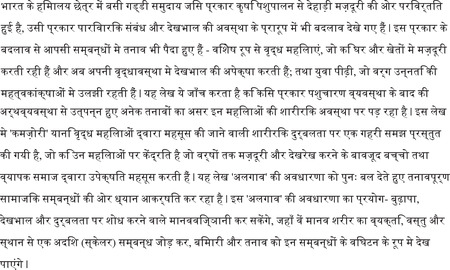

## Introduction

When I first met Skuntala, in the early days of my fieldwork on the southern slopes of the Indian Himalayas, she was working. Methodically, she was cutting the vegetables she had foraged in the foothills that morning with a hooked knife at her feet. Her knees were folded around her ears in the way that only the supple elderly sit, bodies gnarled after years of hard work in the fields. It was impossible to tell if Skuntala was in her 50s or her 80s. Her thin frame still carried extraordinary weight. Rod‐straight, she could balance piles of dung on her head. She wore her hair in a thin plait at the base of her neck and often kept her head covered by a scarf. She didn't wear it in the provocatively demure way of the younger generation of housewives, but tied it practically around her head, keeping it out of the way as she worked. Skuntala rose early to milk the cow and take it to pasture, she attended to breakfast for her sons, then walked over to the riverbank to feed the horses. After lunch, she would go to the old mud house to sow, weed, or harvest until dusk, returning wearied by the sun. Such a regimen of work had sedimented in her body. She would frequently complain of weakness and fatigue. “I am *kamzor* (weak) now,” she would mutter, absently. She seemed unable to engage with the world around her, as if she was lost in translation. Skuntala's daughter explained, “My mother [has] suffered a lot. Now she is tired, she can't really do anything, she can't make decisions anymore, and she is kamzor.”

The word kamzori, as used in Hindi 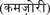, Urdu 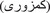, Punjabi 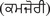, and other South Asian languages, has a more expansive meaning than the English word *weakness*, describing a mode of embodiment that is both social and relational (Varma 2020: 36). It is used across South Asia to describe loss of physical and mental vitality, and depletion of bodily substance (Cohen 1995, 1998; Das 2015; Snell‐Rood 2015; Weaver 2017). Anthropologists, particularly in South Asia, often see such bodily weakness as an expected part of the aging process (Lamb 1997, 2000; Marriott 1998). As a person enters the last stage of their life and nears death, they withdraw from productive work and become more marginal in household work. The weakening of their body parallels the weakening of their attachment to kin and the natural world. Complaints of bodily weakness might even articulate spiritual strength and demands for greater care from younger generations. For the Gaddi tribal women of the Indian Himalayas, with whom I conducted 15 months of ethnographic fieldwork, a benign form of bodily weakness was sometimes enfolded into expected processes of aging. However, kamzori was more often used in a pathological sense to indicate a painful and disrupted process of aging where one was not adequately cared for by kin, not able to rest or withdraw from work. Such a condition cannot, I argue, be understood through existing cultural models of aging, but warrants attention to broader structural transformations that have shaped relations of labor and care.

Indeed, configurations of labor and care have changed dramatically over the lifetime of elderly Gaddis, shifting ideas of personhood, expectations of femininity and notions of bodily vitality. The Gaddi community are a formerly agro–pastoralist Scheduled Tribe who inhabit the foothills of the Dhaula Dhar mountain range in Kangra and Chamba, Himachal Pradesh, India. The increasing unfeasibility of their semi‐nomadic livelihood, as a result of land enclosure, has pushed Gaddi men toward the military, government service, or informal waged labor in the growing tourism, hydropower, slate mining, and construction sectors (Axelby 2007). This shift is paralleled by, first, a transition away from Shaivite animistic religion and toward mainstream Hinduism. Second, there has been a transformation of kinship and marriage structures. The breakdown of the pastoral economy has precipitated the movement from exchange to hypergamous prestige marriage, the nuclearization of the Gaddi household, and renewed emphasis on conjugal intimacy (Kapila 2004; Phillimore 1982). Women were previously significant contributors to household incomes, working in the fields and along the grazing route and taking responsibility for the care of clan gods (Kapila 2022). Today, they are encouraged to occupy themselves with housework and childrearing in order to cultivate Hindu middle‐class respectability. Such shifts introduce tensions between elderly women, who have labored in the house and fields their whole lives, expecting care in old age, and young women, who experience their own pressures of class aspiration, employment, and propriety.

In this article, I take kamzori as a window into the myriad tensions of the post‐pastoral economy and household as they are experienced in the bodies of elderly Gaddi women. Drawing on two contrasting examples of successful and painful aging, respectively, I suggest that kamzori is a bodily materiality that expresses disrupted relations of care between elderly women, their kin, and the wider socio–political milieu. In kamzori, a woman's offering of labor and care over a lifetime is not reciprocated, leaving her bodily substance depleted. I associate alienation as a concept that captures this state of unreciprocated bodily vitality. Alienation, I suggest, might be used by anthropologists studying aging, care, and debility beyond the Gaddi context to envisage the body in scalar relation to people, things, and places, and illness as disruption of such relations.

## Weak and Aging Bodies

Aging is almost always construed as an inevitable but fraught temporal process in both the individual and the social body (Cohen 1994). Specifically, it issues a problem of relational personhood and reciprocal care. How do one's membership, role, and status in society shift over time? How and when should the labor and care one has given be repaid in old age? Bodily changes are seen as signs of aging because of the ways such changes shape expectations of care, personhood, and kinship (Buch 2015: 281). Frailty, debility, and weakness have ambivalent meaning in many contexts, associated sometimes with spiritual strength or authority, and at other times with neglect or moral decay (Cohen 1998; Livingston 2003).

Cultural accounts of aging in South Asia often see bodily weakness as an expected biomoral condition. In the biomoral approach, the body is composed of humours, saps, essences and substances that ebb and flow across the lifecourse, as a result of transactional relations with kin and environment (Alter 1999: S46; Marriott 1976). As a Hindu woman enters old age, she must move from the position of “chief feeder” of the household toward death (Marriott 1998). She must disentangle herself from the web of *maya* (worldly concerns) for which she has been responsible and aspire for other‐worldly return (Lamb 1997). She should be able to rest and enjoy the care of younger generations, replacing her household and productive labor with the bestowal of blessings. She must initiate a bodily self‐cooling process through the food she eats, the clothing she wears, her sexual activity, her expression of emotion (Vatuk 1990). This process of aging is difficult but desirable, such that “denigrating flesh” is seen as a means by which the elderly signify their need for more care and withdraw from household labor (Lamb 2000: 147). Women who are successful in this process take on spiritual strength, where they shed gendered markers of temperamentality, allowing them to take on new forms of masculine or ascetic authority (Wadley 1995).

Biomoral accounts have been critiqued for their overemphasis on cultural representation. In the wish to understand comparable cultural patterns of personhood, diverse life experiences, historical, and political conditions are glossed over (Biehl et al. 2007). As applied to aging, they sometimes hold nostalgic and orientalizing tendencies that present great forces of modernity or capitalism as corrosive to cultural patterns of eldercare (Buch 2015). More recent literature attempts to correct such tendencies by investigating the micropolitical contexts from which complaints of aging arise. Julie Livingston (2003), for instance, shows how the rise of chronic illness in Botswana renders elderhood longer but more fraught, where physical weakness marks social disempowerment rather than aggregated authority, as it once did. For Lawrence Cohen's (1995, 1998) low‐caste Camar interlocutors in Banaras, kamzori in the elderly is associated with senility, *hath pair* (non‐functional hands and feet), impotence in men, and a return to youthful weakness in women. Cohen investigates the ways in which complaints of kamzori are inseparable from complaints of caste, economic, intergenerational, and gendered disparity. People are weak because they are old, but also because they are poor, and because they have “bad families,” Cohen suggests. As such, the condition offers sufferers a way of obliquely speaking about oppressive social hierarchies while preserving their moral integrity.

Cohen and Livingston reveal that bodily weakness is not necessarily desirable, but it is still considered an expected, albeit painful, indicator of aging. To some extent, bodily weakness is expected in the Gaddi aging paradigm, yet I encountered expressions of kamzori in elderly women that appeared pathological or premature. Understanding the acuity of the condition requires attention not only to the cultural or micropolitical context but also to the broader structural transformations in political and domestic economy that shape and disrupt processes of aging. How might we bring into view these different scales of analysis—the disrupted body, the neighborhood or household, and broader structural forces?

Here, I am inspired by an anthropology of care that is concerned with the unstable, contingent networks of capitalism that surround us as they shape intimate life and generate unequal forms of personhood (Bear et al. 2015). Taking the domestic, or the neighborhood “ecology of care” as a point of departure (Das 2015; Gammeltoft and Oosterhoff 2018; Singh 2021; Snell‐Rood 2015), scholars have been able to scale upward to broader politico–economic forces, and downward to intimate, sensory modes of embodiment. This careful scaling reveals that economic and political adjustments manifest in everyday life in ways that are not straightforward and cannot be captured in social diagnoses like the “breakdown of the family” or “rise of consumer society” (Han 2012: 11). As applied to processes of aging, such an approach can reveal how dramatic shifts in political and domestic economies introduce uncertainty into relations of care that might be registered as bodily ailments. For example, Kristin Yarris (2011, 2014) shows how precarious waged or migrant work among younger generations in Nicaragua forces grandmothers to take up new caring responsibilities that leave them with searing headaches (*dolor de cerebro*) and ruminating minds (*pensando mucho*), unable to enjoy the rest or care from their daughters that they believe they deserve. Clara Han (2012) shows how elderly women who have survived the Pinochet regime in Chile are left to care for their grandchildren as their children suffer substance abuse. They register this burden in nervous conditions, as their sacrifice to their kin and nation goes uncompensated. In these cases, we see the elderly mark unreciprocated contributions of labor or care on their bodies in commentaries not only directed at intimate others, but to wider vistas of economy and society. To this literature, I contribute an account of kamzori, and recuperate the concept of alienation to capture forms of non‐reciprocity within these unstable networks.

## Methods

Over 15 months of ethnographic fieldwork (2017–2018), I built a house—embedded in the domestic network of a Gaddi family. My positionality as an unmarried, half‐Indian woman allowed me to be enfolded into structures of care as an adopted daughter. Working with my Gaddi research assistant, I looked outward across the village and surrounding areas through a household, health, and well‐being survey. I further used kinship mapping interviews (*n* = 8) to chart the networks of care and exchange that animate social life within and between households as they have shifted over time. This initial comprehensive survey allowed me to build rapport across caste groups and to identify women who experienced distress, illness, or violence. I layered this with semi‐structured and illness narrative interviews (*n* = 44) with Gaddi women across caste groups (Groleau et al. 2007). I thematically analyzed these interviews and contrasted insights with oral histories provided by Gaddi elders and secondary interviews with nodal figures (doctors, ritual healers, NGO workers, government officials). I drew on archival sources in colonial gazetteers and settlement reports to scale upward from these ethnographic insights to historical processes that shape this community. It is with this deep history that we might begin to understand the changing shape of social and economic life for Gaddi women.

## Historical Context

**Figure 1:**
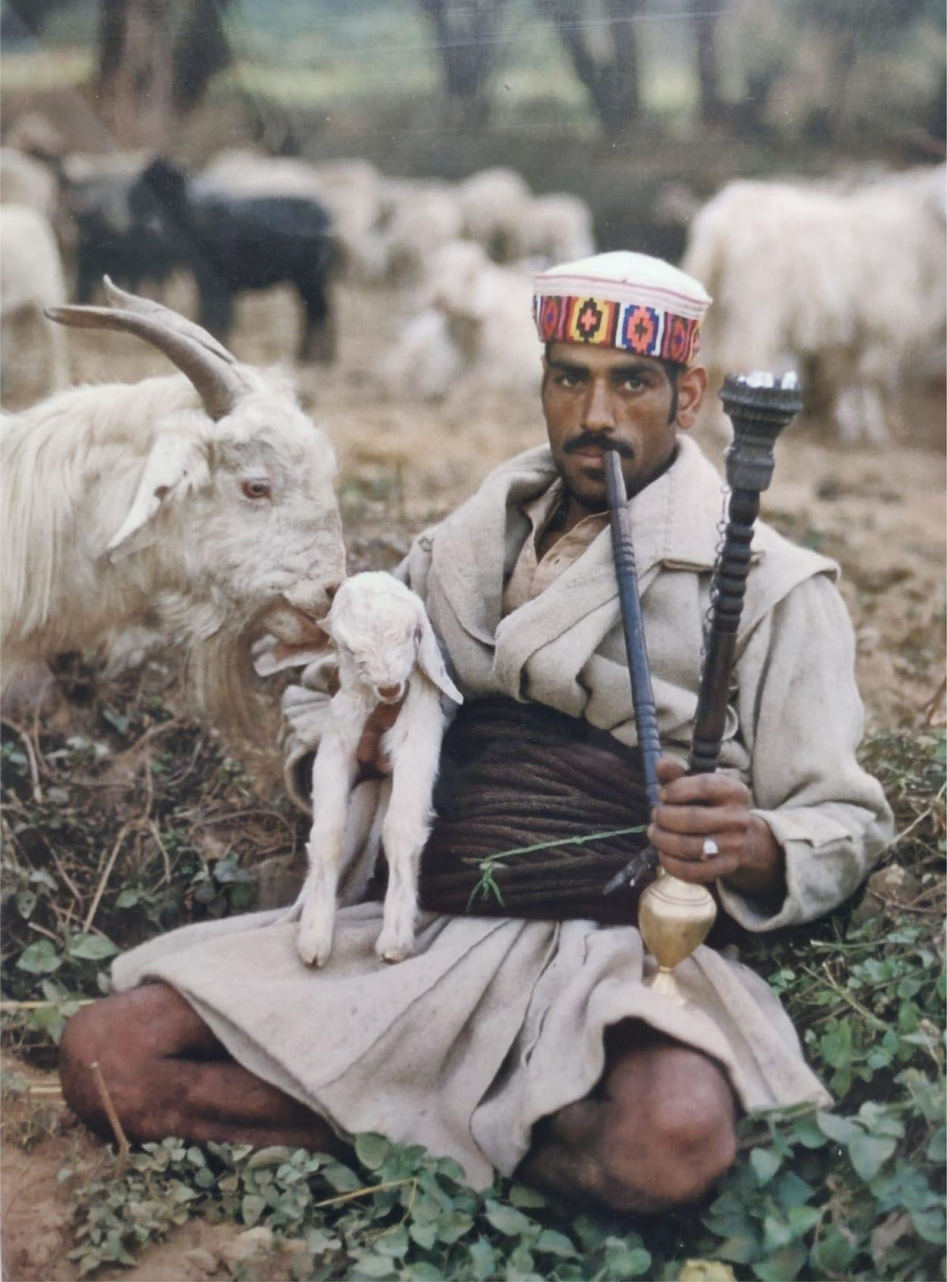
A Gaddi shepherd smoking. (Courtesy of Tejinder Singh Randhawa, ∼1980) [This figure appears in color in the online issue]

Over the past century, agro–pastoralism has become increasingly unviable for the Gaddi people, catalyzed by change in property relations. Prior to the annexation of Punjab by the British Raj in 1846, Gaddi people were granted inheritable rights to access land by local rulers (Kapila 2003). There was no distinction between public, communal, and private land (Axelby 2016). Though marriage was patrilocal and wealth was concentrated in the hands of the head of the flock, the household structure was loose, neither nuclearized nor joint. Families had multiple houses along grazing routes and shepherds often had wives or mistresses in each house, and female infidelity was not necessarily a matter of errant sexuality (Kapila 2003, 2004). Men and women both engaged in agricultural and pastoral labor, business negotiation, and production of wool and meat for market (see Figure 1). The distinctions of gender and caste were less pronounced in the western Himalayan regions than in the plains because local differences of wealth were less pronounced, and women of all castes took a prominent labor role in productive work (Barnes 1855; Berreman 1960; Sharma 1980).

The new perception of proprietorship that evolved following the annexation of Punjab saw the partitioning and privatization of communal lands (Chakravarty‐Kaul 2015; Singh 1998). Grazing land was internalized under village management, while other “wastes” were nationalized and brought under direct government control (Singh 2009: 76). Gaddis found their access to forests closed, their rights redefined, their taxes increased, and the rhythms of their movements controlled (Bhattacharya 1995: 54). The enclosure of common land did not result in an immediate transition but was the catalyst for a century‐long shift in land‐use arrangements, income generation strategies, domestic economy, and moral values.

**Figure 2:**
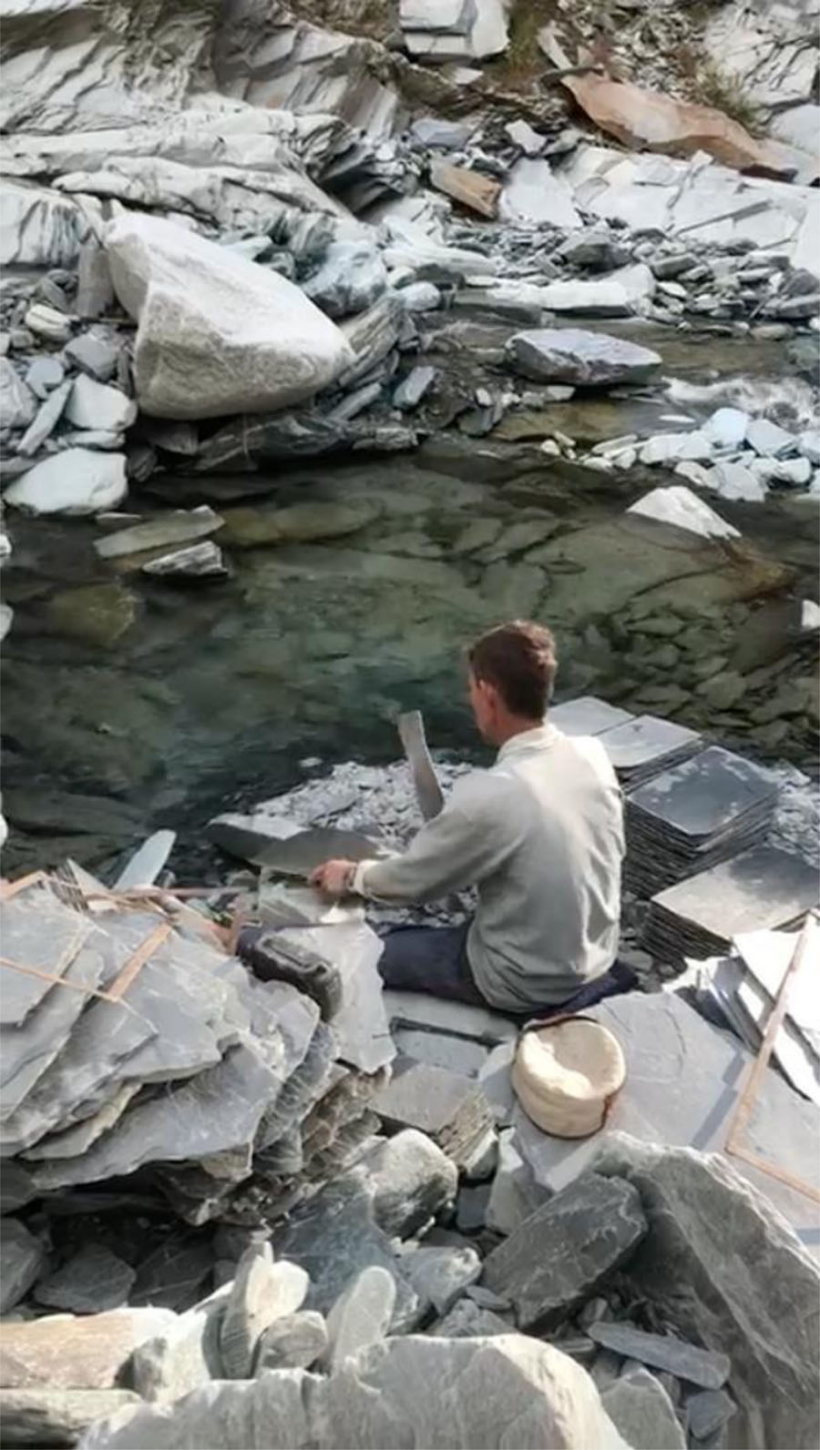
A Gaddi slate miner. (Photograph by the author, 2018). [This figure appears in color in the online issue]

As much as Gaddis were pushed out of pastoralism, they were pulled into other livelihoods favored first by the colonial administration and then by the Indian state. Gaddis began to buy land south of the Dhaula Dhar in Kangra district from the mid‐19th century. Benefits included the opportunity for additional cultivation, access to the waste land that surrounded these holdings for grazing, and the booming industry of slate mining that lined the southern slopes (see Figure 2). By the time of Independence in 1947, the threat to the agro–pastoral economy intensified as slate mining, the lure of state‐provided education, and the dramatic increase in population and habitation in the Kangra valley restricted the land available for either pastoralism or agriculture. Divisions between caste groups within the tribe—high‐caste Gaddi Brahmins, Kshyatria Rajputs, Ranas and Thakurs, and low‐caste Halis, Badis and Sipis—were intensified by endogamous marriage, unequal access to land, and labor opportunities (Christopher 2020; Kapila 2008). These structural shifts in political and domestic economy feature heavily in the personal biographies of elderly Gaddi people.

**Figure 3:**
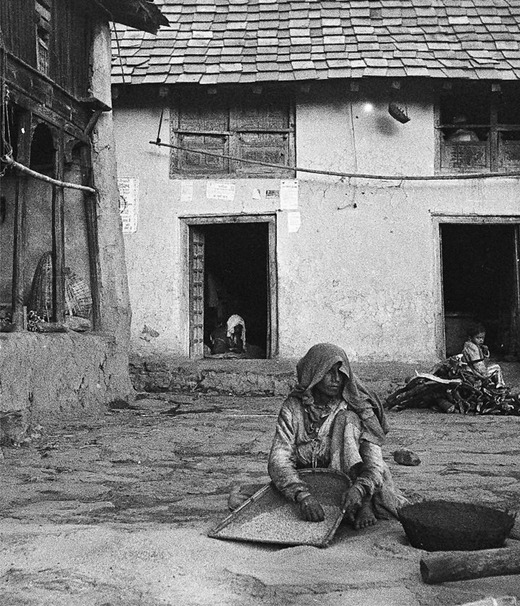
Gaddi woman cleaning wheat, 1977. (Courtesy of Peter Phillimore)

As Gaddis took up sedentary agriculture or waged labor in Kangra, the female contribution to household income became less visible and devalued (see Chowdhry 1994). Where pastoralism required the pooling of household resources, cash cropping or wage labor required the division of productive and reproductive labor. Where men were responsible for generating monetary income, women were responsible for small‐scale subsistence agriculture and reproductive labor. They were no longer instrumental in productive activities such as tending to the flock or spinning wool. As Gaddi people settled in Kangra, it was necessary to align themselves with upper‐caste Rajputs and Brahmins and shed their historical position as dependent laborers. They came face to face with the new kinds of patriarchal norms of their Pahari neighbors (Phillimore 1991). Particularly for Gaddi Rajput men, to secure social prestige it became necessary to exhibit greater authority over their wives and daughters. Normatively, this meant the rise of female seclusion in the home, the practice of *ghungat*—sexual avoidance and veiling in front of one's husband's male relatives—and the withdrawal of women from productive labor. This did not mean women ceased to work altogether, but that they engaged in subsistence agriculture and animal husbandry that came to be considered “housework” (see Figure 3).

**Figure 4:**
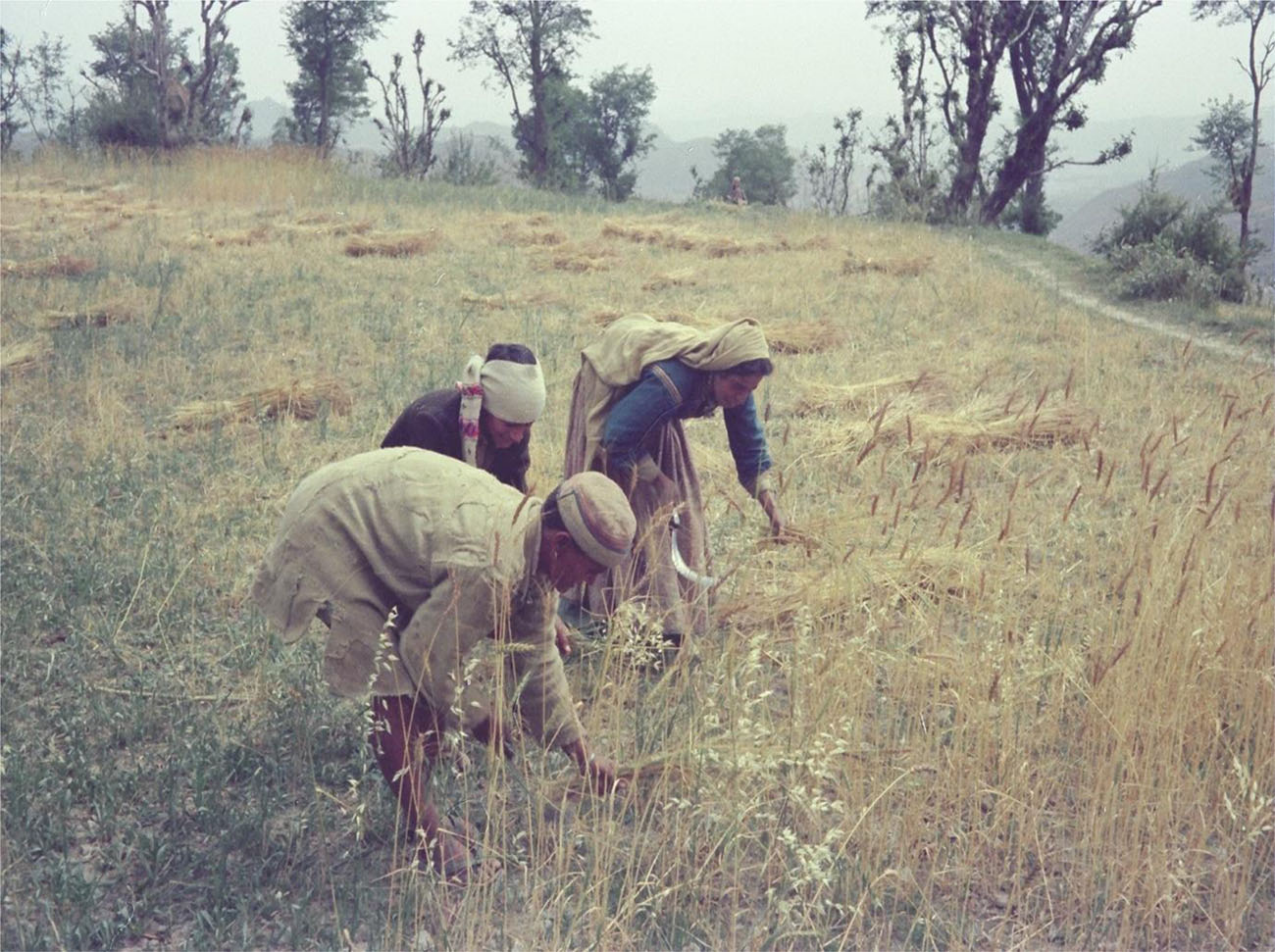
Three Gaddis threshing wheat, 1977. The one in the foreground is a *sadhin*. Peter Phillimore (1991) argues that the emergence of sadhin—Gaddi women who renounce marriage and sexual relations, wear the everyday clothing of Gaddi men and keep their hair cropped short—was a response to increasing anxiety about female propriety and domesticated labor. The social role allowed women to inherit property and grazing permits and to tend flocks in the absence of male heirs. (Photograph courtesy of Peter Phillimore). [This figure appears in color in the online issue]

Increased anxiety about “domesticated”’ female labor and sexuality was codified in the creation of a new kind of Gaddi household and new roles for women, underpinned by a permanent conjugal bond (Kapila 2004) (see Figure 4). Increased inequalities of material wealth and social prestige between Gaddi families animated a more hierarchical marital structure (Newell 1962; Phillimore 1982). Families became nuclearized, defined by blood rather than care, animated by a more rigid division between male as breadwinner and woman as housewife (Kapila 2003). The very question of who counts as a Gaddi was politicized by the access to reservations, where scheduled tribal status was awarded in Kangra in 2002 only to Gaddi Rajput, Thakur, and Brahmin castes and excluded low‐caste Hali (Badi) and Sipi castes, based on their unique lifestyle and shepherding livelihood at the very moment that it was being lost (Christopher 2020; Kapila 2008).

## Loss of *Dharam*

These structural shifts in domestic and pastoral economies have shaped the lives of elderly Gaddi people. They spoke of these shifts as ushering in a deep sense of uncertainty to everyday life. On one hand, the present was seen as better than the past for both individual families and the Gaddi community. The movement away from “backward” traditions meant that Gaddi people were no longer considered poor. For example, one Gaddi elderly man, Rajesh, pointed out the migrant laborers from central India who lived in ramshackle tents on the construction sites that dotted the village. “We will never be like them,” he said, “because we have our own land, and our women don't need to work like that.” Though they remembered hungry, dirty childhoods, they would not struggle for their own children's food.

On the other hand, elderly people lamented the moral decay that had come with a shift in livelihoods. “People these days are terrified, there is more fear now than there has ever been,” another man, Sonki, told me, citing a rise in jealousy, witchcraft, and illness in his neighborhood. When I asked why, he explained that it was a change in *dharam*—or the moral way of life—that had come with a loss of pastoral livelihoods. A third elderly man, Pritam, explained: “Fifty years back, we were the people in the mountains. We were totally self‐sufficient. The women were the keepers of this dharam.” *Dharam* bound together concerns of health, well‐being, gendered, and casteist respectability, domestic organisation, connection to landscape and animals. It was achieved through particular forms of labor, or *kam*.^1^ Elderly people were most concerned with a loss of self‐sufficiency that came with the breakdown of dharam. “We were the kings of the mountain,” an elderly man cried. Their narratives lament younger generations’ dependency on the state and market. Jagdish, a fourth Gaddi elderly man explained:

I am speaking about one hundred years ago, now we have had freedom [from the British] for 70 years. I am 72 years old. I am talking about things that have happened in my lifetime. Our ancestors were uneducated. … Mountain people ate simple food and made woollen things to cover themselves. … People had to work to fill their stomachs. There weren't any special government schemes to provide work. But now it is a new scientific age, and everyone has left that old lifestyle—the young generation is all studying. Now they are doing learned jobs and will earn 70–80,000 [rupees] per month, they will eat, and they buy everything. They will not make their own things. They are forgetting their culture. They depend on others.

Most importantly, elderly people's narratives indicated a broad‐reaching critique of younger generations carelessness. One Gaddi woman explained: “People want short cuts. They don't care about each other anymore. They want to do things quickly these days.” Men were especially concerned with younger women's lack of respectability and eschewal of patriarchal authority. This was explained by Jagdish:

In the new generation, the [mental] changes that have happened to girls have been caused by the control of society. We and our society try to control them and put pressure, because they aren't able to live like we did in the old times. We want them to live with *izzat* (honor, respectability). We lived like that because we feared our parents and respected them. In those times, people were very uneducated and respected each other. When they are all educated, they become more cunning. Girls particularly, they want their own things. But if we're not able to give them those things, then they'll run away. They think that if they live like us then they will become *dafar* (useless).  If they live in their own way, then we'll become dafar.

By contrast, elderly women were more concerned with the obsolesce of their *kam*. They drew attention to the physical hardship that they had suffered through their lives, hardship that was not experienced or honored by upwardly mobile younger generations. This hardship was acute for elderly widows or women whose husbands had not secured profitable land, military, or formal employment, but instead engaged in precarious waged work as laborers or slate miners. Parvana, a Gaddi widow reflected that when her children were young her husband died suddenly, and there was no way of getting resources for the family. “I had to go and work in people's houses, then after that go to the fields to work. While I was working, I had to watch over the children, so I had to take them with me … the children's lives were bad, and my life was bad.” But now, Parvana said, her children don't experience this kind of precarity, because of her hard work they were able to go to school and earn an income in good jobs instead of having to work for others or do farming. Parvana was proud of this achievement, but she also felt that it was not acknowledged.

Elderly Gaddi women's anxieties about younger generations tended to focus on their daughters and daughters‐in‐law. They were concerned that their sons marry educated women to improve their status, while fearing their daughters‐in‐law would not continue to perform dharam's domestic, agricultural, and ritual labor as they aspired to middle‐class domestic practices or tertiary education, or pursued business opportunities. Young women even took on different forms of ritual labor—neglecting the care of traditional household gods, and instead engaging in mainstream Hindu practices such as *karwa chauth*, a day of fasting and prayer for their husbands, or praying to Hindu gods like Ganesh who were unheard of in generations before. Reena, a Gaddi woman in her 70s, chuckled cynically at the thought of fasting for the “useless, drunken fool” that was her husband, and giving up a day of work. The discontinuity of ritual labor had great significance, as it was traditionally a woman's role to transmit female substance to future generations through worship of household gods (Kapila 2022). Elder women often failed to appreciate the financial and social strains on young women, caught up instead in exchanges on social media, watching soap operas, and always “running back to their natal home.” They were piqued that younger women didn't acknowledge the hardship that previous generations had gone through to accumulate wealth and respectability.

## Loss of Substance

Elderly people's anxieties relating to the loss of dharam were often discussed through bodily change. For this generation, the landscape was the primary source of vitality, and their bodies were considered to consist of the substances drawn from that place (Wagner 2013). Health was largely associated with having good blood (*khun*), which in turn comes from compatible foods grown on the land, grounded in a caste‐based theory that buttresses the ideology of descent (Parry 1979). Many spoke of the times before, when Gaddi bodies had strength, fitness, and agility as a result of *kam—*communion with the landscape and animals. People ate the fresh, nourishing meat of goats grazed on flowers and herbs, as well as home‐grown seasonal vegetables, and they drank healing goat's milk. They had been able to climb the mountains with ease, to resist the cold, wind, and rain.

With sedenterization, the rhythms of Gaddi bodies have changed, and the authoritative claim to the landscape has been diluted. Most waged work is not considered by elderly people to be a source of vitality. Elderly women lamented that their daughters were unable to even climb to the high hills above the village to cut grass for their animals, as their bellies had become swollen by daily consumption of rice. Habituation to heating foods like alcohol had spoiled blood and demeanor, leaving people with overt passions, lust, and aggression. These qualities were said to result in alcoholism and violence for men and sexual impropriety for women. The body was further spoiled by the consumption of vegetables from the market, ruined by dependence on the “poisoned” foods sprayed with pesticides that are imported from Punjab. Such foods were the cause of new kinds of ailments unseen before, including kidney and gall stones, forms of cancer and heart disease, disrupted digestive processes, and jaundice. Elderly people who could still feed their families from the fresh meat of their own herds and home‐grown vegetables saw themselves as healthier and morally superior to their neighbors.

This generalized state of bodily disruption caused by the loss of dharam was the backdrop against which more acute complaints of kamzori were made. Elderly people expected to experience kamzori in the later stages of aging. However, I found that it was commonly used to indicate premature and pathological loss of bodily substance, especially in women with grown children. These women described kamzori as a loss of *sakti—*or feminine power*—*and *takat—*physical strength. It was marked by feelings of exhaustion and lethargy. Bodily, it was signified by wasting muscles, weight loss, joint pain, back pain, “low BP” ( low blood pressure), disrupted digestion, stomach pain, and appetite loss. It rendered the body resistant to nourishment and medication. Some of its psychological symptoms included insomnia, hallucination, and rumination. Kamzori could lead to weakness of mind, leave the boundaries of one's body permeable and render one vulnerable to attacks of black magic (*opara*), and witchcraft (*jadu*). Kamzori could also lead to opportunistic illnesses such as fevers and heart problems.

Kamzori was not experienced prematurely by all elderly women. It was more likely to be felt by those residing in households that were fraught with tension or conflict, or by women who had experienced significant hardship and hard work over their lifetimes, such as widows. Men who experienced fraught domestic situations were more likely to directly blame their kin for inadequate care or respect. By contrast, women who did not receive such care from kin seemed to use kamzori to obliquely express anxieties around generational and spousal responsibilities for care. One Gaddi woman, Juganoo, who had supported her husband with the herd all her life, complained of kamzori and searing headaches that appeared around the time she was trying to find her son a suitable bride. Another, Rinky, became afflicted with kamzori and jaundice (*piliya*) when her son and his wife migrated to Delhi to take up waged work, leaving her alone and unable to manage her household. A third, Sarita, explained that she had become weak, bedbound and “soft‐minded” soon after her daughters‐in‐law had “abandoned” her and became consumed with her own small tailoring business. A fourth, Rani, explained that she was kamzor because her husband had become an alcoholic, leaving her to bring in money by working in a local construction site. Indeed, expressions of kamzori were not used to directly blame husbands or younger generations, but to render visible forms of discontent directed both at kin and at wider structural milieu.

## Two Aging Women

I turn now to two comparative cases: Jagatambo, a woman who was able to navigate this fraught present and did not experience kamzori; and Skuntala, a woman who was unable to do so, and experienced a painful and premature kamzori.

Jagatambo was an upper‐caste Gaddi widow who was installed in the palatial house of her son, a successful property dealer. I met Jagatambo when I was in the midst of interviews with other widows of the village, weighed down by tales of financial insecurity. In contrast, Jagatambo welcomed me up freshly tiled stairs to an ornate balcony where she was having tea with her two daughters‐in‐law. Jagatambo sat peacefully as I asked her about the loss of her husband some 10 years prior. “It was normal,” she explained, “my children were there to help me and give me money.” She explained that she had stopped wearing red, her favorite color, and begun to dress simply, but she still ate mutton and chicken even though this wasn't customary for widows. “Mummy is the head of the house still!” Her daughter‐in‐law, Radha, chuckled affectionately. Jagatambo could rest as she had a doting daughter‐in‐law who still tended fields of wheat and corn, cooked with home‐grown ingredients, and carried out daily prayers for the household gods. Jagatambo was able to replace her material contribution to the household with the bestowal of blessings. “My children are always there for me,” she said when I asked who supported or cared for her. “I have thousands of friends as well.” She spoke particularly of her dear friend who lived in nearby Palampur and mentioned that they were able to visit one another or speak regularly over the phone.

Midway through our interview, Jagatambo's son Surjeet entered, wearing a well‐ironed shirt and thick gold chain. Jagatambo's husband had been a shepherd, and she had even supported him on the shepherding route throughout her married life. When he died, Surjeet had sold the family's whole herd of sheep and goats for six lakhs and bought land in a Kangra village. The family gave up their semi‐nomadic life and settled permanently so the younger members could go to school. Surjeet quickly sold this land for much more than he had paid for it and began a thriving property business. Sweeping his hand across the horizon, he showed me that he still kept a great deal of land for subsistence and cash cropping. The women of the house, though dressed now in expensive silk suits, helped sow and harvest these crops and kept a cow for fresh daily milk—honoring the wishes of their mother‐in‐law.

Jagatambo herself was able to withdraw from such work. She described her body as aging, but such descriptions appeared benign, resonating with the expected process of enfeeblement indicated in cultural accounts of South Asian aging. Her contribution to the household might be increasingly obsolete, but her authority and wishes were still honored by her son, who provided financially, and her daughters‐in‐law, who were both caring and submissive. Such removal from labor was difficult for elderly women who, unlike Jagatambo, didn't trust the younger generation to secure the vitality of the household or provide adequate care. It involved stepping away from the lifetime of work they had done to build, sustain, and reproduce a household. This work was exhausting, but it was also foundational to their sense of self. Let us turn now to Skuntala's much more painful account of aging.

Unlike Jagatambo, when upper‐caste Gaddi couple Skuntala and Jagdish settled in Kangra with their three children, they did not give up their flock. Skuntala has spent her life on the shepherding route with her husband, but their lives were marked by a steadily increasing sense of hardship as the mountains filled up. As the hardships of shepherding grew for Jagdish, and he responded by turning to alcohol, so did the hardships of the household for Skuntala. Her skills became increasingly obsolete. There was no need to go with her husband and the herd, for Jagdish kept only 50 goats. There was no need to spin coarse wool into yarn to be sold at the market, for Tibetans bought their wool from cheaper Rajasthani goats. There was no need to grow subsistence crops when they could be bought subsidized by the government “Below Poverty Line” card they were entitled to because of their scheduled tribe status. The bulk of the household income came from their children. Their daughter was married to a military man. Their youngest son had a steady job as a hospital peon and was on his way to completing his own MA in computers. Even their eldest son, after eight years of shepherding with his father, gave that up to begin his restaurant.

During my fieldwork, Skuntala and Jagdish were trying to find a bride for their eldest son, Raman. As an eminent Rajput family, they sought a match with an educated girl who would be able to elevate the class status of the family. But finding a bride proved difficult. It was rumored that Jagdish's shepherding work put many girls off. Finally, a bride was found in Meena, the youngest daughter of a wealthy Gaddi businessman. Jagdish poured his savings into constructing a new concrete house for his son and secured a sizable loan for the wedding.

After the wedding, Skuntala left Meena alone, allowing her to adjust to the new household. She moved into the new concrete house but looked anachronistic among the modern appliances. Often unable to sleep in the raised beds, she would curl up by the fire in the kitchen. Skuntala kept returning to clean the family's mud house each day, unaccompanied by her sons or daughter‐in‐law. It became clear that Meena was not interested in helping Skuntala tend to the horses, cut the wheat, or climb the foothills up to the family shepherding hut. Meena complained of a sore back and locked herself in her plush room, spending hours on the phone talking to her sisters. Skuntala did not chide her, but instead resigned herself, crestfallen. Meena was concerned with securing a position with a local tailor so that she could earn some money herself. As the daughter of a businessman, she had never done the hard fieldwork or trekking that this family was used to. Her family no longer worshipped a household god, so she looked blankly when Skuntala explained how she worshipped a clan god instead of going to the Hindu temple.

Skuntala's complaints of kamzori grew acute when the atmosphere of the household was particularly fraught due to tensions in her marriage or her relationship with her daughter‐in‐law. She described the condition as a chronic sense of bodily depletion that caused her to lose her strength. She also feared that it left her vulnerable to *opara*—witchcraft or black magic. Sometimes, surrounding moments of family conflict, her condition crescendoed into fever, insomnia, or muscle ache. For instance, in the height of summer, when the family had been living in the concrete house for some six months and Meena's disinterest in agricultural work became apparent, Skuntala began to spend more and more time in the old mud house. She developed a high temperature. I lay beside her as she described feverish hallucinations of grassy, flower‐filled fields. She complained that she couldn't sleep in the new concrete house. She told me “I am thin and kamzor, and since I have moved to this new [concrete] house I have been ill. I haven't had any health problems in my life. But now I just have this kamzori.” When Skuntala's condition was at its worst, I tried to take her to the hospital. In the cavernous corridors, she looked even more disoriented. “My main problem can't be solved here in the hospital because I know what it is. It is kamzori.” She waved her hand dismissively—the condition, she told me, rendered her body resistant to medication. Still, when she was feverish, she went to the fields to work; she said it was only through such work that she might find her strength again.

## Alienated Bodies

When women like Jagatambo received the wealth, care, and respect that they thought they deserved in these new domestic and economic conditions, they did not tend to experience premature symptoms of kamzori and were able to age peacefully. Here, successful aging was associated with strong relations of care within the household and a retention of domestic authority, as well as with the ability of an elderly person to benefit from the present circumstances of market integration, such as land possession, prestige marriage, or wealth accumulation. Indeed, the ability of younger generations to honor the livelihoods and wishes of their elders was, to some extent, dependent on the wealth they gained outside the pastoral economy. Intergenerational relations were facilitated by favorable economic circumstances and the willingness on the part of younger generations to rework traditional ideas of Gaddi respectability into patterns of elderly care.

In contrast, for women like Skuntala, who did not receive such care, respect, and vitality from kin, complaints of kamzori and its associated ailments proliferated. These forms of bodily weakness were not blamed directly on family members or the loss of domestic authority, and sufferers did not attribute distress directly to vast forces like urbanization. Instead, the condition expressed more subtle tensions in domestic relations of care and a more diffuse anxiety about the devaluation of women's labor, conditions of housing, ritual piety, their children's employment prospects, or the challenges of the marriage market. For example, even though the acute symptoms associated with Skuntala's kamzori flared up during family conflict and surrounding Meena's behavior, she attributed the condition to the concrete house and the general hardship she had experienced over the course of her lifetime. It was not Meena's specific refusal to work in the fields or to come to the shepherding hut that necessarily offended Skuntala, but a more general melancholic sense that such work was not valued by a younger generation, and hence that Meena did not appreciate the contribution that Skuntala had made to the household by honoring her wishes.

Here, care for the elderly is not only physical care or even respect, but a wider ethics of acknowledgment, attentiveness, and appreciation of the mundane sacrifices that have been made over a lifetime. As Maya Mayblin observes among Santa Lucian women in northeast Brazil who have experienced great hardship, this sacrifice is not easily located in classic anthropological terms because, rather than constituting an event, it is more of a lived aesthetic—a generative mode of being‐in‐the‐world (Mayblin 2014: 357). In kamzori, this sacrifice is marked on the body as a depletion of bodily substance, substance that should be provided through such care or acquired through this increasingly obsolete dharam and kam. As such, women did not seek rest, medication, or biomedical care in kamzori, but instead tended to do more work in the fields or the household in an attempt to recuperate lost vitality. Women like Skuntala returned endlessly to sow fields that their families were no longer dependent on for flour, and tended cows that their families were no longer dependent on for milk. Culturalist accounts of South Asian aging gloss over the affluence and stability of economy and household that makes successful aging possible. So, too, might micropolitical analyses of expected bodily weakness be augmented by deeper analysis of the structural processes that disrupt processes of aging.

Indeed, kamzori resembles complaints of weakness in the ethnographic record that are not necessarily associated with aging, but with wider fractured relations of domestic and political economy. Snell‐Rood's (2015) interlocutors, women living in a Delhi slum, cite kamzori that resulted from emotional and physical endurance in conditions of urban poverty. Rashid (2007) points out that adolescent married women living in Bangladeshi informal settlements also speak of the weakness (*durbolota*) that an excess of caring responsibilities embedded in their bodies. For Varma's (2020) interlocutors—psychiatric patients in Kashmir—kamzori rendered visible the psychic impact of occupation that could not be treated as trauma and persisted in the presence of biomedical care. Beyond the South Asian context, Taussig (1992) observes a white working‐class woman suffering a condition of progressive muscle deterioration resultant from poor medical care and abysmal conditions of poverty. In Gaddi kamzori and these accounts, pathological bodily weakness allows sufferers to articulate a deficit of care and a lack of acknowledgment from intimate others. The voicing of such concerns is the first step toward healing distressed relationships. The act of telling kamzori initiates new relations of listening, borrowing, visiting, often between women, that work to relieve distress (Han 2012: 38; see Stack 1974). Such telling works not to assign blame but offers a commentary on wider strains in changing domestic, political, and economic milieu. I suggest that this scalar, relational constellation of illness might be well conceptualized through alienation.

Alienation has been used in a loose and ill‐defined way by medical anthropologists to describe how aetiologies of suffering are displaced from the state or structure to the individual through medicalized language (Farmer 1997; Taussig 1992). This use of alienation is heavily influenced by Marxist thought, where it is the condition of distorted self‐consciousness experienced as a result of the conditions of labor that characterize the capitalist system. The experience of this distortion is embodied and psychological: “[capitalism] squanders human lives … and not only blood and flesh, but also nerve and brain” (Marx cited in Blackledge 2013: 50). For example, Scheper‐Hughes draws attention to the condition of *nervoso* in Brazilian cane workers as an alienation of body and mind, “a collective delusion such that the sick‐poor of the Alto can … fall into a mood of self‐blaming that is painful to witness” (1992: 195).

While the impulse to understand how structural conditions are experienced in the body is commendable, there is a lack of precision in the attribution of illness to broad structural forces condensed down into individual bodies (see Singh 2021). Such accounts tend to patronizingly assume suffers’ own accounts of illness aetiologies are false consciousness, or focus only on the alienation of mind from body, rather than the ways that people are alienated in a wider sense from social relations. A Maussian notion of alienation, while indebted to Marx, proves more productive to think with as it is untethered from class relations (Mauss 1990). A Maussian notion of alienation is premised on alienated or unreciprocated relations between persons and things, and might be seen as a shadow of the “dividual” or relational personhood that has been so influential in the anthropology of South Asia. Put simply, people are bound to each other and to the things that surround them by durable links that provide a personal identity for the individual (Carrier 1992: 539). Here the work of building relations with people, things, and the landscape signifies an expansive and ethnographically variable notion of labor as social creativity (Graeber 2001)—effecting the world through the creation of new social forms and relations—framed by local cosmological idioms of productivity and vulnerability (Turner 2008). Alienation is a state in which these open‐ended relations are not reciprocated and thus ruptured, leaving the giver *alienated* from that which they have given.

Bodily weakness, in the pathological sense, might be seen as a form of alienation. In the Gaddi case, shifts in livelihoods and domestic economies leave expectations of care and labor deeply uncertain for elderly people. Elderly women are denied adequate care or respect from their kin and cannot trust them to secure the vitality of the household by following dharam or doing kam. The sacrifice of labor that elderly women have given over their lives goes unacknowledged and unreciprocated, as waged work and middle‐class lifestyles become desirable. This fractures the expected process of aging and leaves elderly women's lifetime contribution to the household obsolete for younger generations. This state of unreciprocated care is articulated as depleted bodily substance, premature kamzori. Hence, expressions of kamzori speak to the tensions of gender and generation in the household, but also to wider conditions of politico–economic change.

Framing such a state as alienation allows us to see the multiple levels at which relations of care are fractured. Indeed, kamzori allows suffers to insist on the relationality of their own bodies and render visible the ways in which such relations, with kin, governments, landscapes, and medical systems are non‐reciprocal. Alienation might be used by anthropologists of care, aging, and debility to capture specific histories of structural transformation where relations between people, things, and the environment are unreciprocated, having psychic and embodied effects. Framing relational illnesses like kamzori through the lens of alienation allows us to envisage, first, the ways in which structural conditions shape economies of labor and care and are thus experienced intimately in the body. Second, it shows how people scale upward from their individual bodies, through tense intimate and domestic relations to comment on inadequate structural conditions through illness and distress. Here I contribute to ongoing discussions in the feminist anthropology of care that attempt to correct a romantic and unthinking assumption that care might always be equated with good feeling (Berlant 2016). We see how women use their aging bodies to express the uncertainties and strains of care.

## Coda: Finding Relief

For Skuntala, relief from kamzori was only truly left in one place—the shepherding hut where she spent her monsoon summers. It was only here, she said, that her feelings of kamzori would leave her. “After we plant the corn, we will go back there. It is such a beautiful place, it is our place, so peaceful,” she said. Sure enough, she left for the hut as soon as the corn was in the ground. I went to visit her in late October, only a few months before my departure from Kangra. I arrived at the hut late in the evening, as the sun was setting. The hut was set high in the mountains, on the last ridge before the tree line that broke up into the high passes. Skuntala and Jagadish's careful work was evident in the curated interior, such that the room held a dated dignity. The hearth was painted smooth with a fresh layer of dung paint. Skuntala cooked dinner, slicing marrow she had harvested that morning. As she worked, she was completely absorbed, so much so that she couldn't hear when I called to her softly. She absently cried “*jai*” under her breath, “*jai Mata, jai Shiva, jai Ram*” [praise the Mother, praise Shiva, praise Ram]. After we ate the marrow around the fire that evening, Skuntala lay down beside by me. Her hollow cheeks were slightly fuller. As she lay there, she smiled her gummy smile. “How do you like our place?” She asked tenderly. I said many times that it was so beautiful. She smiled more widely, content. She spoke of how she would bring her children to this hut in their childhood, how her daughters would climb up here in one hour, loaded with big sacks of supplies, where now they can't even make it to the slate mines. She spoke of the passing of time, of her hard life, wearily but with acceptance. That night we slept soundly, swaddled in thick blankets. The next day she sent me home with a bag of corn, a bottle of goat milk, and a pot of vegetables for her son, which I found again days later, untouched in the fridge of the new concrete house.

## Note

1
*Kam* has no particular etymological relation to kamzori.
